# Technology Integration in Syrian Medical Education From the Perspective of Students and Faculty: A Cross-Sectional Evaluation

**DOI:** 10.2196/76958

**Published:** 2025-08-08

**Authors:** Subhiya Hassoun, Mayssoon Dashash, Adnan Baddour

**Affiliations:** 1 Medical Education Department, Syrian Virtual University, P.O. Box 35329, Damascus, Syrian Arab Republic; 2 Department of Pediatric Dentistry, Faculty of Dental Medicine, Damascus University, Damascus, Syrian Arab Republic; 3 Faculty of Pharmacy, University of Kalamoon, Deir-Attia, Syrian Arab Republic

**Keywords:** technology integration, technology-enhanced learning environments, blended learning, medical education, faculty members

## Abstract

**Background:**

Technology-enhanced learning (TEL) has become increasingly vital in global medical education, offering significant advantages in knowledge acquisition, communication, motivation, and student engagement. In Syria, a country facing prolonged crises, there is an urgent need to evaluate the integration of technology within medical education to address institutional limitations and support student learning.

**Objective:**

The aim of this study is to evaluate the awareness, perceived challenges, and needs regarding the integration of technology in medical education from the perspectives of students and faculty at Syrian medical colleges.

**Methods:**

A cross-sectional survey was conducted during the 2023‐2024 academic year across Syrian universities. Stratified random sampling was used to recruit 500 medical students and 200 faculty members. Two tailored, self-administered questionnaires were used, covering motivation, perceived benefits, challenges, and suggestions for technology integration. Validity was assessed through expert review and pilot testing (n=30), and internal consistency was confirmed (Cronbach α=0.6‐0.7). Quantitative data were analyzed using descriptive statistics, *t* tests, ANOVA, and Kruskal-Wallis tests.

**Results:**

Among medical students, 94% (470\500) agreed that integrating technology into medical education is essential, with similar agreement from 93.5% (187\200) of faculty. No significant differences were found in student responses based on specialization (*P*=.32) or university type (*P*=.11). Likewise, faculty perspectives did not significantly differ by academic qualification or years of experience (*P*>.05). There were several perceived benefits; for instance, 93.2% (n=466) of students reported that technology kept them up to date with new developments, 88% stated it enhanced research skills, and 86.8% found TEL more enjoyable than traditional learning methods. Most respondents (95% n=475) said TEL created a flexible, interactive environment. Among faculty, 77% (n=154) agreed TEL improves clinical skill development. Respondents noted there were some challenges; specifically, 57% (n=285) of students cited poor internet service, 33% (n=165) noted the financial burden, and 82.2% (n=411) called for behavioral guidelines. Among faculty, 85.5% (n=171) cited lack of institutional support and 90% (n=180) emphasized the need for training. Both groups supported the development of communication platforms, curriculum revisions, and faculty development programs.

**Conclusions:**

There is a strong consensus among Syrian medical students and faculty on the value and necessity of integrating technology in medical education. Despite infrastructure and administrative challenges, both groups recognize TEL as a powerful tool for improving clinical competencies, student motivation, and academic engagement. Institutional commitment, curricular reform, and tailored training are essential to achieving sustainable, effective technology integration.

## Introduction

The landscape of medical education is rapidly evolving in response to the exponential growth of medical knowledge, the demand for lifelong learning, and changing societal expectations regarding professionalism, patient safety, and evidence-based care [1,2]. Technology-enhanced learning (TEL) has emerged as a key strategy to meet these demands by offering flexible, interactive, and student-centered educational environments. TEL methods—including web-based modules, virtual simulation, and mobile learning—are shown to improve knowledge retention, clinical reasoning, motivation, and engagement across various health disciplines [3-5].

Globally, blended learning models that combine face-to-face instruction with web-based components have gained widespread acceptance, particularly after the COVID-19 pandemic highlighted the need for resilient educational systems [6,7]. In the context of medical education, TEL fosters not only cognitive and technical skill development but also collaboration, self-directed learning, and emotional engagement [8]. These advantages make TEL an essential component of modern medical curricula, particularly in low-resource or crisis-affected settings.

In Syria, however, technology integration into medical education remains underexplored. The country’s prolonged conflict and resulting humanitarian crisis have severely impacted higher education institutions, contributing to gaps in infrastructure, access, and pedagogical innovation [9,10]. Despite these challenges, there is growing interest in leveraging TEL to improve teaching and learning outcomes in Syrian universities. Initiatives such as web-based modules, digital assessments, and virtual classrooms have gained traction, though systematic evaluations remain limited [11].

Recognizing the gap in technology integration within Syrian medical education, this study aims to provide a comprehensive formative evaluation of the extent to which TEL has been incorporated in this context. By examining and analyzing the perspectives of both medical students and faculty members, the study seeks to assess current levels of awareness about TEL, identify institutional challenges and requirements, and generate practical, actionable insights to enhance its implementation. Understanding stakeholder perceptions is essential for developing culturally appropriate and context-sensitive strategies that support innovation and resilience in Syrian medical education. This formative approach goes beyond purely descriptive assessments; this study presents a formative evaluation of technology integration in Syrian medical education. By examining the perspectives of both medical students and faculty members, this research aims to inform future educational planning and policy reform in Syrian higher education. The study’s findings can guide the development of support systems, capacity-building programs, and infrastructure investments aligned with both local realities and global standards in medical education.

## Methods

### Study Design

A cross-sectional study was conducted across multiple public and private medical colleges in the Syrian Arab Republic during the winter semester of the 2023‐2024 academic year. The participating institutions included faculties of medicine, dentistry, pharmacy, and health sciences affiliated with universities located in major Syrian cities such as Damascus, Aleppo, Latakia, and Homs.

### Study Population

The study population consisted of key stakeholders involved in the integration of technology into medical education at Syrian universities. This included faculty members and educational personnel with PhD degrees, master’s degrees, and other degrees in various fields including human medicine, pharmacy, dental medicine, and health sciences. The sample accounted for years of experience and included both academic and administrative staff, regardless of their decision-making roles. Additionally, medical college students were included, taking into consideration their respective colleges, year of study, and whether they attended public or private universities.

Eligibility criteria for students included enrollment in one of the targeted colleges during the academic year, while faculty participants were required to be actively employed at the time of data collection.

Given the diverse nature of the study community, stratified random sampling was used. The population was divided into two homogeneous samples from which random samples were drawn. The number of participants (n) in each sample was proportional to the size of the stratum (N).

### Survey Design

The study used a self-administered anonymous questionnaire, and a systematic approach was used to ensure equal representation within the targeted sample, including only those participants who completed the questionnaires.

Two questionnaires were designed, each tailored to a specific group from the study population. The questionnaires were created in Arabic on the basis of prior studies related to the topic, field observations, and expert consultations. An English version of the questionnaire is available in MultimediaAppendices 1  2. A 5-point Likert scale, ranging from 1 (strongly disagree) to 5 (strongly agree), was used to assess attitudes related to study variables.

Questionnaire A was designed for sample A to evaluate medical college students’ awareness of the technology integration process at Syrian universities. Demographic data such as gender, specialization, academic year, and university type were collected. This questionnaire consisted of 23 items divided into 4 sections. The first section consisted of 7 items focused on evaluating motivation to learn in TEL environments. The second section included 7 items exploring skills and experiences gained in TEL environments. The third and fourth sections discussed the challenges and needs associated with the technology integration process in medical education, comprising 9 items. Additionally, an open-ended section was included for participants to provide further recommendations or suggestions regarding technology integration in medical education (Multimedia Appendix 1).

Questionnaire B was directed toward sample B to assess the awareness of educational members in medical colleges at Syrian universities. It gathered information on academic specialization, qualifications, and years of experience. This questionnaire included 24 items divided into 3 sections. The first section consisted of 11 items aimed at analyzing the respondent’s personal point of view on the goal of technology integration in medical education. The second and third sections consisted of 13 items focused on the requirements and challenges faced during the integration process, alongside an open-ended field for additional recommendations or proposals related to technology integration in medical education (Multimedia Appendix 2).

### Data Collection

Participant recruitment was conducted through a combination of direct email invitations, university announcements, and social media channels used by academic institutions (eg, WhatsApp groups, Telegram channels). Interested individuals were provided with an explanation of the study purpose, ethical approval details, and a secure Google Forms link to the relevant questionnaire.

Participation was entirely voluntary, and responses were collected anonymously to ensure privacy and minimize response bias. Only fully completed questionnaires were included in the final analysis. A total of 500 students and 200 faculty members completed the surveys, yielding a 100% response rate based on the targeted sample.

### Ethical Considerations

This study received ethical approval from the ethical research committee of the Syrian Virtual University (number 2258/0, dated August 8, 2024). The research was conducted in compliance with the Declaration of Helsinki and relevant national ethical standards.

All participants were informed about the objectives of the study and their rights as participants. Participation was voluntary, and informed consent was obtained electronically before the questionnaire was administered.

Participants’ confidentiality and anonymity were strictly maintained. All data were anonymized and securely stored in password-protected files accessible only to authorized research team members. The data are available upon request from the corresponding author.

This study did not involve any clinical interventions or procedures. It consisted solely of a self-administered questionnaire assessing perceptions and experiences of faculty and students regarding technology integration in medical education.

No images or supplementary materials in this study contain identifiable information or visual representations of individual participants. No identifying personal data were collected, and all responses remained anonymous. No compensation was provided to participants in this study.

### Instrument Development and Validation

Two separate, structured, self-administered questionnaires were developed to capture the perspectives of medical students and faculty members regarding the integration of technology into Syrian medical education. The instruments were created in Arabic, drawing on existing literature, institutional observations, and expert consultations in medical education and educational technology. English translations are available in MultimediaAppendices 1  2.

The questionnaire for students (Sample A) consisted of 23 items across four domains: (1) motivation to learn in technology-enhanced environments, (2) skills and experiences gained, (3) perceived challenges, and (4) institutional needs. The faculty questionnaire (Sample B) contained 24 items across three domains: (1) attitudes toward technology in medical education, (2) perceived requirements, and (3) challenges. Both instruments included open-ended items for qualitative input. Face and content validity were established through a panel of 5 medical education experts who assessed each item’s relevance, clarity, and alignment with study objectives. Based on their feedback, items were revised for clarity and cultural appropriateness.

A pilot test was conducted with 30 participants (15 students, 15 faculty members) from Syrian universities who were not part of the final sample. Respondents provided structured feedback on questionnaire clarity, response burden, and item comprehension. Minor adjustments were made accordingly.

Internal consistency reliability was assessed using Cronbach α. For both instruments, α values ranged from 0.6 to 0.7 across all domains, indicating acceptable reliability for exploratory research in social and educational contexts [4]. All items used a 5-point Likert scale ranging from 1 (strongly disagree) to 5 (strongly agree).

### Sample Validity and Reliability Test

Reliability is a critical characteristic of a study tool. The importance of measuring the reliability lies in obtaining consistent results. The reliability of the overall questionnaire was verified via the Cronbach α coefficient, which ranges from 0 to 1, with an acceptable threshold of 60% in social studies. The reliability coefficient values for the study sections ranged from 0.6 to 0.7, indicating acceptable reliability and suggesting that the questionnaire is valid and reliable.

### Data Analysis

To meet the study objectives, the data were transferred from Google Forms to Microsoft Excel, and the Statistical Package for the Social Sciences software (version 25; IBM Corp) was used to analyze the final data. Descriptive statistics, including the mean (SD), were calculated. Statistical methods were used to analyze the data, test the study hypotheses, and describe the participants’ responses. Quantitative variables are reported as mean (SD), whereas qualitative variables are reported as frequencies and percentages. A 5-point Likert scale was adopted, with the mean calculated for each statement (out of 5), each section of the evaluation, and the total questionnaire. Mean values and ranges were used to provide a verbal interpretation of the mean responses: strongly disagree=1.00‐1.8, disagree=1.81‐2.60, not sure=2.61‐3.40, agree=3.41‐4.20, strongly agree=4.21‐5.

For Sample A, an independent sample *t* test was used to determine significant differences in skills and experiences gained in TEL environments according to the type of university. The Kruskal-Wallis test was used to determine significant differences in motivation to learn in TEL environments on the basis of the specialization variable. A significance level (*P*<.05) was established to determine statistical significance.

For Sample B, an independent sample *t* test was used to determine significant differences in the personal opinions of faculty members in medical colleges, regardless of whether they held administrative positions (decision-makers). A 1-way ANOVA test was used to determine significant differences in faculty opinions regarding technology integration in medical education on the basis of years of experience, whereas the Welch test was conducted to determine significant differences in faculty opinions about technology integration on the basis of the academic degree. A significance level of *P*<.05 was applied to determine statistical significance.

### Qualitative Data Analysis

Responses to open-ended questions were analyzed using a basic thematic content analysis approach. After initial reading, the responses were coded and categorized into emerging themes. The analysis was conducted manually by two researchers independently, who then discussed and resolved any discrepancies to ensure consistency. Thematic saturation was assessed informally, and illustrative quotes were selected to represent common viewpoints and highlight participant insights.

## Results

### Sample Analysis

Sample A consisted of 500 medical college students from Syrian universities who met the criteria and voluntarily agreed to participate in the study (Table 1). Among the participants, females represented 61.2%, whereas males accounted for 38.8%. In terms of university affiliation, 37.2% attended governmental universities, whereas the majority, 62.8%, attended private universities. The highest percentage of students by specialization was among dental students (35.6%), followed by pharmacy students (32.4%) and medical students (32%). The largest group by academic year was fifth-year students at 25.6%, followed by second-year students at 20.6%, fourth-year students at 19.6%, third-year students at 18%, and first-year students at 16.2%.

**Table 1.** Demographic characteristics of Syrian medical students (N=500) and faculty members (N=200) who participated in a cross-sectional study on technology-enhanced learning conducted during the 2023‐2024 academic year.

Characteristics		Values, n (%)
**Sample A (students)**		
***Gender ***		
	Male	194 (38.8)
	Female	306 (61.2)
***University ***		
	Governmental	186 (37.2)
	Private	314 (62.8)
***College ***		
	Medicine	160 (32)
	Pharmacy	162 (32.4)
	Dentistry	178 (35.6)
***Year of study***		
	First	81 (16.2)
	Second	103 (20.6)
	Third	90 (18)
	Fourth	98 (19.6)
	Fifth	128 (25.6)
**Sample B (faculty)**		
***Specialization ***		
	Human medicine	57 (28.5)
	Dental medicine	65 (32.5)
	Pharmacy	49 (24.5)
	Health sciences	29 (14.5)
***Academic degree***		
	Bachelor’s degree	74 (37)
	Master’s degree	85 (42.5)
	PhD	41 (20.5)
***Administrative position ***		
	Yes	20 (10)
	No	180 (90)
***Years of experience***		
	Less than 5 years	80 (40)
	5-10 years	79 (39.5)
	More than 10 years	41 (20.5)

Conversely, Sample B included 200 faculty members from medical colleges at Syrian universities (Table 1). The largest percentage came from the faculty of dentistry (32.5%). This was followed by specialists in medicine (28.5%), pharmacy (24.5%), and health sciences (14.5%). With respect to academic qualifications, 42.5% held a master’s degree, 37% held a bachelor’s degree, and 20.5% held a PhD. In terms of professional experience, 40% had less than 5 years of experience, 39.5% had 5‐10 years, and 20.5% had more than 10 years. Additionally, 10% of the faculty members were decision-makers and held administrative positions.

### Statistical Analysis

All participants completed the questionnaire, resulting in a 100% response rate. The overall evaluation of the questionnaires was positive as indicated in Table 2 (the English versions of the questionnaires are provided in MultimediaAppendices 1  2). Most participants agreed that integrating technology into medical education is necessary today, with a mean score of 4.5 for sample A and 4.4 for sample B. Furthermore, participants from both samples agreed that TEL environments enhance communication skills among students, with mean scores of 3.6 for this statement. The participants reported challenges and requirements associated with the integration of technology, with a mean score of 3.25 for sample A and 3.79 for sample B for the entire section (Table 2). We also obtained the mean Likert-scale responses from medical students and faculty members regarding motivation (students: 4.02), skills (students: 4.08; faculty: 3.04), challenges (students: 3.25; faculty: 3.62), and needs related to TEL (students: 3.50; faculty:3.79) in Syrian medical education.

**Table 2.** Descriptive statistics for items evaluating technology-enhanced learning Results are based on responses from students (Questionnaire A) and faculty (Questionnaire B) at Syrian medical universities.

Statement evaluation and item or section number		Mean	Standard deviation	Expressions
**Questionnaire A**				
***Section 1***				
	1.1	4.5	0.6	Strongly agree
	1.2	4.4	0.5	Strongly agree
	1.3	4.3	0.7	Strongly agree
	1.4	3.3	1	Not sure
	1.5	4.1	0.8	Agree
	1.6	4.3	0.7	Strongly agree
	1.7	3.3	1	Not sure
***Section 2***				
	2.1	4.3	0.6	Strongly agree
	2.2	3.9	0.9	Agree
	2.3	3.8	0.9	Agree
	2.4	3.6	1	Agree
	2.5	4.22	0.7	Strongly agree
	2.6	4.4	0.6	Strongly agree
	2.7	4.4	0.6	Strongly agree
***Section 3***				
	3.1	3.06	1.1	Not sure
	3.2	3.5	1.1	Agree
	3.3	3.01	1	Not sure
	3.4	3.45	1	Agree
***Section 4***				
	4.1	4	0.7	Strongly agree
	4.2	3.4	1.1	Agree
	4.3	3.3	1.1	Not sure
	4.4	3.6	1.1	Agree
	4.5	3.2	1.2	Not sure
***Section evaluation***				
	S.1	4.02	0.7	Agree
	S.2	4.08	0.7	Agree
	S.3	3.25	1.05	Not sure
	S.4	3.5	1.04	Agree
**Questionnaire B**				
***Section 1***				
	1.1	4.4	0.6	Strongly agree
	1.2	3.14	1.2	Not sure
	1.3	3.9	0.9	Agree
	1.4	2.9	1.1	Not sure
	1.5	3.7	0.9	Agree
	1.6	3.9	0.8	Agree
	1.7	3.6	1.08	Agree
	1.8	2.7	1.05	Not sure
	1.9	4.2	0.7	Strongly Agree
	1.10	3.8	1.02	Agree
	1.11	3.8	0.8	Agree
***Section 2***				
	2.1	3.09	1.2	Not sure
	2.2	4.28	0.8	Strongly agree
	2.3	4.29	0.7	Strongly agree
	2.4	4.39	0.7	Strongly agree
	2.5	3.6	1.07	Agree
	2.6	3.09	0.9	Not sure
***Section 3***				
	3.1	4.19	0.8	Agree
	3.2	4.18	0.7	Agree
	3.3	3.9	0.9	Agree
	3.4	3.48	1.19	Agree
	3.5	3.22	1.1	Not sure
	3.6	2.9	1.14	Not sure
	3.7	3.53	0.9	Agree
***Section evaluation***				
	S.1	3.64	0.9	Agree
	S.2	3.79	0.8	Agree
	S.3	3.62	0.9	Agree

Independent sample *t* test showed no significant difference between the dependent variable and the independent variable regarding the skills and experience gained in TEL environments among students from governmental (mean 4.0845, SD 0.47542) and private universities (mean 4.1306, SD 0.53298; *P*=.11; *P*<.05). When comparing motivation to learn in TEL environments on the basis of students’ specialization, the Kruskal-Wallis Test was applied after confirming the test hypotheses and conditions, ensuring the normal distribution of the sample, and validating it through the QQ plot. The results revealed no significant differences, as the average responses for motivation to learn in TEL environments were similar. Medicine students had an average score of 264.23, dental students had an average score of 247.13, and pharmacy students had an average score of 241.22, with a *P* value of .32. These findings are presented in Table 3. Similarly, no significant differences were observed when comparing faculty members’ opinions about TEL environments on the basis of years of experience and academic degree, according to the results of a 1-way ANOVA and Welch test (Table 4).

**Table 3.** Kruskal-Wallis test comparing student motivation to learn in technology-enhanced learning environments across academic specializations (medicine, dentistry, pharmacy) among students in Syrian medical colleges.

College	Mean	SD	*P* value
Medicine	4.136	.5307	.32
Dentistry	4.068	.5446	
Pharmacy	4.028	.5753	

**Table 4.** One-way ANOVA and Welch test comparing faculty members’ perceptions of technology-enhanced learning environments based on academic degree and years of teaching experience in Syrian medical institutions.

Variable	Mean	SD	*P* value
**Academic degree**			.22
Bachelor	2.6462	.92423	
Master	2.6298	.62768	
PhD	2.5543	.69019	
**Years of teaching experience**			.92
Less than 5 years	2.5352	.77935	
5-10 years	2.6974	.70759	
More than 10 years	2.6364	.81616	

### Student Preferences for Learning Environments

Among the 500 surveyed students, preferences for learning environments varied notably. We found that 47.2% (n=236) of students preferred traditional, in-person instruction, while 18.6% (n=93) favored TEL environments. The remaining 34.2% (n=171) expressed a neutral stance, indicating no strong preference for either mode.

These findings suggest that while TEL is recognized as beneficial, a substantial portion of students still favor traditional approaches, possibly due to familiarity, infrastructure limitations, or insufficient exposure to well-implemented digital tools.

### Perceived Challenges of TEL

**Figure 1.**
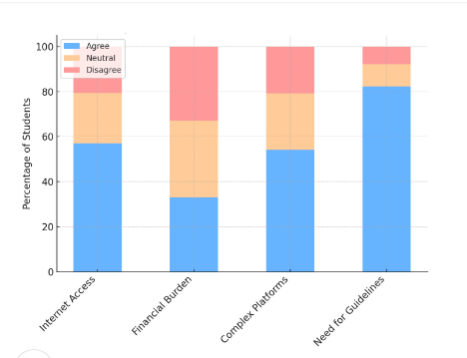
Percentage of students facing challenges in technology-enhanced learning enviroments in syrian medical educationn.

Figure 1 shows the most commonly reported challenges faced by students when engaging in TEL environments. The majority 57% (n=285) cited poor internet access as a major barrier, followed by the need for behavioral or usage guidelines 82.2% (n=411), and difficulty navigating complex platforms 54.2% (n=271). Financial burden was also a concern, with 33% (n=165) of students reporting cost-related challenges. These findings highlight persistent infrastructure and support issues that may limit the effective adoption of TEL in Syrian medical institutions.

### Qualitative Analysis

Open-ended responses provided further insight into participants’ perspectives on technology integration. Thematic analysis revealed several recurring themes among both students and faculty members. These themes reflected practical needs, perceived barriers, and suggestions for improvement in TEL environments. Table 5 summarizes the most frequently mentioned themes along with representative anonymized quotations.

**Table 5.** Summary of qualitative themes derived from open-ended responses regarding technology-enhanced learning in Syrian medical education, including frequency and representative quotations from students and faculty.

Theme	Frequency, n	Sample quotation
Need for digital training	62	“We need proper training on how to use new platforms.”
Lack of institutional support	48	“Our college has no clear policy for using technology.”
Internet connectivity issues	85	“The connection is so bad I often miss online lectures.”
Motivation from interactive tools	51	“Videos and interactive quizzes make learning enjoyable.”

## Discussion

### Principal Findings

This study examined the perceptions of medical students and faculty members regarding the integration of technology into medical education across Syrian universities. The findings revealed high levels of agreement from both groups on the importance and value of TEL. Students and faculty identified similar benefits—such as improved clinical skills, communication, and flexibility—as well as common challenges, including poor internet access, institutional limitations, infrastructure and administrative challenges, and lack of training. No significant differences in perceptions were found based on demographics such as university type, specialization, or faculty experience. However, there was a strong consensus among Syrian medical students and faculty on the value and necessity of integrating technology in medical education, and both groups recognized TEL as a powerful tool for improving clinical competencies, student motivation, and academic engagement. Institutional commitment, curricular reform, and tailored training are essential to achieving sustainable, effective technology integration.

### Interpretation and Comparison With Prior Studies

#### Perceptions of Technology Integration and Its Impact on Skills and Learning

This study was designed to evaluate the current situation of technology integration into medical education at universities in Syria via a self-administrated questionnaire. A total of 500 medical students from medical colleges (medicine, dentistry, and pharmacy) and 200 faculty members completed the questionnaire regarding their knowledge and awareness of the technology integration process and its applications in medical education.

The findings indicated a high level of awareness and attention among participants regarding the importance and necessity of TEL in medical education. Specifically, 94% of medical students and 93.5% of faculty members agreed on the necessity of integrating technology in medical education, despite the challenges posed by the current situation in the country. However, no significant relationship was found between students’ specialization and their perception of motivation in TEL. Additionally, there was no significant relationship between faculty members’ academic qualifications or years of teaching experience and their perceptions of TEL environments. A positive trend was observed in agreement across all sections of the questionnaire, likely reflecting a growing awareness among faculty members regarding the need to shift away from traditional and outdated teaching methods in favor of modern methods.

In examining the significance of TEL environments, it is crucial to analyze their impact on the acquisition of cognitive and clinical skills. The results revealed that 77% of faculty members and 66.6% of medical students agreed that TEL environments facilitate the development of better clinical skills. Furthermore, 92.6% of the students held a positive attitude toward the role of technology in enhancing their understanding of course materials, whereas only 39.5% of the faculty members shared the same opinion, with 24.5% remaining neutral and 36% disagreeing. Additionally, 86.2% of medical students stated that technology aids them in their understanding and review of lessons, and 93.4% agreed that it provides access to a wide range of educational resources. Furthermore, 93.2% indicated that technology enables them to stay up to date with new trends in their field of study. These results align with a study on the effectiveness of the gastroenterology e-module for undergraduate medical students at Syrian universities, which concluded that e-learning significantly enhances knowledge levels, with no significant difference between synchronous and asynchronous methods, and good satisfaction and self-confidence among all participants [11]. Another study that evaluated the impact of a web-based restorative dentistry course on learning outcomes reported similar findings, indicating high satisfaction rates and improved understanding of restorative dentistry [5]. A third similar study of a web-based course regarding dental pain management and local anesthesia found that it effectively enhanced students’ knowledge in these areas [12].

Additionally, 86.8% of the students agreed that the educational process is more enjoyable when technology is present, and 95% affirmed that integrating technology into education creates a flexible learning environment that facilitates interaction and skills acquisition. However, 47.2% preferred traditional learning environments, perceiving them as clearer and easier to navigate than TEL environments, which they still find complex and difficult to access. Furthermore, 34.2% of students remained neutral on this issue. Moreover, 54.2% acknowledged the challenges of engaging in TEL environments, attributing this to various difficulties, barriers, and requirements.

#### Communication, Engagement, and Educational Value

Although 33% of faculty members expressed concerns that TEL environments may diminish the role of the teacher, 75.5% agreed that these environments enhance student-centered learning, and 46% believed that they promote individual differences among students. Moreover, 58.5% agreed that these environments enhance communication skills. Additionally, 59.4% of medical students felt that technology enhances their communication skills, and 64% believed that it allows them to express their opinions freely and without hesitation. Furthermore, 88% reported that technology has helped them develop their research skills, whereas 84.2% stated that it encourages their continuous medical education. Faculty members also recognized the positive impact of technology across these dimensions, with agreement rates ranging from 75% to 86%.

A recent publication evaluating the effectiveness and feasibility of an asynchronous web-based course aimed at improving the skills of Syrian health professionals in evidence-based medicine reported that the course was highly effective, fostering positive attitudes toward continuous medical education training programs [7].

#### Requirements and Institutional Challenges

The requirements and challenges encountered by the sample members during the integration of technology were highlighted to identify appropriate solutions for overcoming these issues. For example, 33% of medical students agreed that TEL environments impose a financial burden on them, while 57% noted the inadequacy of sufficient and favorable internet services for using these methods, with 22.4% remaining neutral. Furthermore, 82.2% emphasized the need for behavior guidelines to regulate these environments, and 87% highlighted the urgent need for educational communication platforms tailored to each university. Such platforms would facilitate the exchange and accessibility of scientific materials at any time and from any location, as well as enhance communication with professors and peers for sharing information and experiences.

Concerns about requirements and challenges expressed by faculty members showed a high level of agreement; the study indicated that 85.5% of faculty members recognized the urgent need for institutional support at all levels to optimally implement blended learning in universities. Additionally, 85% emphasized the importance of support from department heads, deans, and decision-makers. Moreover, 90% agreed on the necessity of training and developing faculty members’ skills. Notably, 75% of the faculty members identified a lack of teaching experiences as a challenge. A recent study exploring the lived experience of Syrian dentists enrolled in a web-based course on traumatic dental injuries revealed that the course was effective overall, with participants highly motivated to benefit from it despite personal and technical problems and challenges related to the crisis [13].

#### Curriculum Reform and Stakeholder Role

Our study revealed strong agreement among participants regarding the urgent need to revise curricula to align with the rapid growth of medical knowledge and changing health care environments, as well as with the societal expectations of graduates. These findings are consistent with a study conducted among undergraduate medical students at Ain Shams University in spring 2023, which assessed knowledge and attitudes toward artificial intelligence (AI) and its applications in medical education and practice. The study revealed that medical curricula must evolve to adequately prepare students by providing a comprehensive understanding of AI, including its applications, potential, limitations, and ethical considerations, ensuring students are well-prepared for their future careers [14].

Furthermore, 87% of the participants acknowledged the inadequate infrastructure and services within medical colleges. Additionally, 56.5% noted the insufficient time allocated for lectures to use technology and modern techniques. These findings align with a study aimed at identifying the difficulties faced by the Faculty of Basic Education of Mustansiriya University regarding modern education and the use of technology in teaching. This revealed obstacles faced by faculty members, such as inadequate infrastructure, insufficient training on the use of modern educational technology, and a lack of material and moral incentives and suitable lecture halls [15]. Conversely, no significant differences were found in personal opinions about integrating technology in medical education among faculty members, regardless of their administrative roles or years of experience. Similar findings were reported in a study of faculty members at Al-Ahliyya Amman University, which revealed no significant differences in term of gender, college, academic rank, or years of experience [16]. On the other hand, medical curricula evolve alongside advancements in medical practice, and steps should be taken to integrate technology into the medical education curricula. Both medical faculty and students must understand the concept and implantation of technology integration to maximize its utility.

Responses to the open-ended questions concerning students’ opinions about the requirements and challenges of integrating technology in medical education reflected the collective findings from the faculty opinions. One student said that “most teachers present the subject in a narrative, non-interactive manner, without considering presentation methods that engage students.” Another student added, “it is very important to provide appropriate technologies for learning purposes in universities.” A third student emphasized that “medical education is a very precise field and using technology (when applied correctly) will facilitate both theoretical and possibly clinical studies.” A fourth student expressed concerns about the prevalence of technology in medical education, stating “there is an issue with the appropriate use of technology in the learning process; it should be used in a way that avoids monotony and boredom.” Another student expressed that “integrating technology into medical education should be a requirement, not an option, for universities and relevant authorities.” Regarding barriers, one student said, “the difficulty lies in the often poor internet connectivity, and there is insufficient infrastructure for using technology in education.” Another student called for “updating our curricula to keep pace with modern medicine,” while another stressed “it is important to have email access for students and faculty to inquire about academic subjects, communicate with professors, and ask about curriculum, study dates, and quizzes.”

#### Policy Context and National Strategy

Ministers of higher education are striving to create educational environments that meet the needs of modern societies by implementing strategies that combine technology-enhanced learning with traditional methods. Over time, traditional learning is expected to diminish as students progress through their academic years [17]. Additionally, there is an emphasis on developing models that assist educators in their teaching programs and provide frameworks for obtaining accreditation for technology-enhanced learning programs, along with standards to maintain high quality [18].

Despite various challenges such as inadequate infrastructure, limited technical capabilities, problematic virtual learning environments, and insufficient technical skills [9], there is a long-term commitment in higher education to support students and enhance their engagement with faculty members, who are continuously seeking to create effective learning activities that produce competent graduates. However, meaningful technology integration cannot be initiated or supported if the institution lacks knowledge, self-efficacy, pedagogical beliefs, and culture conductive to technology integration [19]. When stakeholders’ perspectives shift to acknowledge that effective learning requires the appropriate use of assistive technology resources, critical milestones will be achieved in ensuring quality as a means of assurance, accountability, monitoring, and enhancement [18]. Moreover, various aspects related to TEL, such as the quality and reliability of learning management systems and the availability of resources, rely heavily on feedback and verification of student learning and activities [18].

#### Solutions and Future Work

Our findings are consistent with global trends in the adoption of educational technologies across medical curricula. A range of modalities can complement traditional instruction and address the specific needs and barriers reported by students and faculty in this study. Technology integration in education leverages digital tools to enhance instruction, collaboration, and professional skill development. Mobile learning, powered by smartphones and apps, helps students build digital fluency through video, audio, and storytelling-based platforms [20], supporting the development of communication, clinical, and problem-solving skills [21]. Social media platforms such as Twitter, Facebook, and YouTube foster interactive learning environments, enabling students to create content, receive feedback, and engage with educational communities [22,23]. Messaging and collaboration tools like WhatsApp, Telegram, and Wikipedia contribute to real-time communication and team-based performance tracking [21]. Flipped classrooms, which involve reviewing video lectures before class [24], transform traditional instruction into active, discussion-based sessions. These in-person sessions often use case-based or team-based learning strategies [25-27], turning the classroom into a dynamic environment for applied learning. Simulation-based learning further complements this approach by offering a safe, controlled space to develop clinical, communication, and decision-making skills [28-31]. Simulations also allow learners to practice rare or complex scenarios, building confidence through experiential methods.

Emerging technologies such as wearable devices and immersive simulations provide hands-free access to instructional content, reduce errors, and support procedural training [32,33]. Virtual and augmented reality enhance engagement and empathy by allowing students to experience clinical and ethical situations in realistic ways, especially in fields like geriatrics and end-of-life care [6,34]. To ensure the safe adoption of these technologies, verification of users’ professional identities is essential [35]. AI is also reshaping medical education, offering personalized content delivery and real-time feedback through AI-based tutoring systems [36,37]. However, integrating AI into curricula requires both educator readiness and the thoughtful design of content to ensure meaningful and ethical engagement with the technology [8].

Financial support and infrastructure improvement are essential to facilitating technology integration. Institutions should consider establishing dedicated classrooms, well-equipped laboratories, display devices, and simulation facilities to enhance learning outcomes [38]. These improvements align with findings that robust infrastructure, funding, and technical support are crucial for successful educational technology implementation in higher education [39]. To establish the goals of medical education, providing sufficient financial support and improving infrastructure and services to facilitate the use of technology in medical education are crucial. This includes the creation of dedicated classrooms and well-equipped laboratories, as well as display devices and medical simulation equipment. Additionally, having specialized technicians available to address issues and specialized programmers to support university e-learning platforms is essential to ensuring the easy accessibility of educational materials. Enhancing communication skills through the exchange of opinions and ideas, discussing cases via the chat feature, and simplifying communication with teachers are also vital. Moreover, deploying specialized technicians and programmers to manage e-learning platforms and troubleshoot technical issues ensures easy access to educational materials, consistent with best practices recommended in systematic reviews [39]; dedicated units and offices are urgently needed at each university to monitor, assess, and evaluate the process of integrating technology into education. These units should provide detailed reports and evaluations of all completed operations for inclusion in the university’s annual report, which may be submitted to relevant authorities as needed. Training courses for faculty, seminars for stakeholders, and documentation of staff tutorials on the integration process should also be organized. Collaborating with companies that specialize in educational technologies is important, and research should be conducted to create tools, standards, and frameworks for performance assessment, which can be adopted to ensure the quality of higher education. Additionally, further studies are needed to evaluate the impact of modern technologies on the quality and development of higher education in key areas including students, curricula, teacher performance, and college/university administration [40].

Effective technology integration requires a deep understanding of its potential advantages and challenges, as well as adequate training and substantial support and incentives for both educators and learners [8]. Importantly, these technologies should not be viewed as replacements for “real-world” medical educators; instead, they should be considered additional tools for learning [35]. However, it is evident that integrating technology in medical education will take time and require adaptability to rapidly changing technologies [4]. Despite the current situation in Syria, the optimal effectiveness of technology integration in medical education presents a significant opportunity for medical students to overcome educational circumstances and enhance their learning skills, even amid economic, social, and health difficulties, ultimately saving time and effort for both students and educators.

### Limitations

This study has several limitations that should be acknowledged. First, the data were collected through self-reported questionnaires, which may be subject to response bias or social desirability effects. Second, although stratified sampling was used to ensure representation from different universities and disciplines, the findings may not be generalizable beyond the Syrian medical education context. Third, while the inclusion of open-ended questions provided some qualitative insight, the study did not use in-depth qualitative methods such as interviews or focus groups, which could have offered a deeper understanding of the participants’ experiences. Additionally, because data collection relied on digital platforms, individuals with limited internet access or lower digital literacy may have been underrepresented. These factors should be considered when interpreting the results and applying them to broader contexts.

### Conclusion and Broader Implications

As technology integration becomes increasingly common in both medical education and health care, equipping future doctors with digital competencies is critical to enhancing clinical outcomes, improving system efficiency, and fostering lifelong learning. This study provides evidence that TEL not only motivates medical students but also significantly improves their engagement, communication skills, clinical competencies, and access to current scientific knowledge. TEL fosters collaboration, supports research, and helps students remain up to date in a rapidly evolving field, making it essential for the training of future-ready physicians. Interestingly, the study revealed no significant correlations between demographic variables (such as student specialization or university type or faculty academic rank or teaching experience) and attitudes toward TEL. This suggests that literacy and acceptance of TEL may be more strongly influenced by exposure and hands-on experience than by traditional academic hierarchies or roles, indicating a broad, cross-demographic readiness for technology adoption in medical education. To translate this readiness into meaningful reform, institutions must commit to developing well-supported e-learning platforms, revising outdated curricula, and shifting pedagogical strategies toward student-centered approaches. Building sustainable TEL environments will require institutional investment in infrastructure, ongoing faculty development, and monitoring systems that assess both learning outcomes and community needs.

Importantly, TEL should not be seen as a replacement for traditional education, but rather as a complementary and empowering tool for both learners and educators. Future efforts must include rigorous follow-up studies to evaluate the long-term impact of TEL implementation and inform adaptive strategies that ensure continuous quality improvement. Despite the economic and logistical challenges faced in Syria and similar contexts, the findings of this study offer a hopeful foundation for the effective and scalable integration of technology into medical education.

## Supplementary material

10.2196/76958 Multimedia Appendix 1 Evaluating technology integration in Syrian medical education (student questionnaire).

10.2196/76958 Multimedia Appendix 2 Evaluating technology integration in Syrian medical education (faculty questionnaire).

## Abbreviations

AI: artificial intelligence
TEL: technology-enhanced learning
