# Supplementary material for: Technology Integration in Syrian Medical Education From the Perspective of Students and Faculty: A Cross-Sectional Evaluation
Source: JMIR Form Res. 2025 Aug 8;9:e76958. doi: 10.2196/76958 (PMC12334109; doi:10.2196/76958)
Supplement: Multimedia Appendix 1 [file formative-v9-e76958-s001.pdf]

A questionnaire evaluating Technology integration in Syrian medical education from the perspectives of medical college students

Dear student,

Integrating technology in medical education aims to create an integrated educational system that seeks to provide educational content using modern techniques and Internet, which helps the student or learner interact with the educational material at the time, place, and speed that suits him and his circumstances and abilities, and takes into account individual differences among learners, This questionnaire was prepared for research purposes and aims to evaluate Technology integrating in medical education at Syrian universities from the perspectives of medical college students.

We inform you that by completing this questionnaire you are totally agreeing to be part of our research, we also confirm that your participation is completely voluntary, and The information collected will be treated with complete confidentiality and will only be used for the purposes of the study. Please, write and tick where appropriate…

We are grateful for your cooperation...

Gender:
o Male o female

Specialization:
o Human medicine o Dentistry o Pharmacy

Academic year:
o first o second o third o Fourth o Fifth

University
o Governmental o Private

section.1	Motivation to learn in Tel environments	Strongly Agree	Agree	Neutral	Disagree	Strongly Disagree
	1. Integrating technology in medical education is necessary nowadays					
	2. Integrating technology in education helps create a flexible learning environment					
	3. Educational process is more enjoyable with the presence of technology					
	4. Traditional learning environments (lectures) is more preferred compared to technology-enhanced learning environments*					
	5. Technology-enhanced learning environments encourage continuous medical learning					
	6. Technology-enhanced learning environments enable me to develop my research skills					
	7. Technology-enhanced learning environments seem difficult to be engaged in					
section.2	Competencies and skills gained in TEL environments	Strongly Agree	Agree	Neutral	Disagree	Strongly Disagree
	8. Using technology tools in learning helps me understand the course material better					
	9. Using technology tools in learning helps me master clinical skills					
	10. Technology-enhanced learning environments enable me to express my opinions					
	11. Technology-enhanced learning environments enhance communication skills among students					
	12. Technology-enhanced learning environments allow me to review lessons easily					
	13. Technology-enhanced learning environments allow me to access a wide range of references and educational resources					
	14. Technology-enhanced learning environments enable me to keep up with advancements in my field of study					
Section.3	challenges face Technology integration	Strongly Agree	Agree	Neutral	Disagree	Strongly Disagree
	15. Integrating technology in learning methods does not align with our curriculum					
	16. Internet service not always available to participate effectively in learning activities					
	17. Technology-enhanced learning environments impose a financial burden on me					
	18. Technology-enhanced learning environments seems difficult to understand the educational materials					
Section.4	Requirements of Technology integration	Strongly Agree	Agree	Neutral	Disagree	Strongly Disagree
	19. Behavioral rules needed to control technology-enhanced learning environments					
	20. Technology tools (screens, computers) are provided to facilitate effective engagement in the educational process					
	21. University provides e-learning systems easy-to-access and use					
	22. University learning platform enables me to access educational materials anytime and anywhere					
	23. University learning platform enables me to communicate and interact with teachers and colleagues					
	If you have any other recommendations or suggestions related, please write it down: ………………………………………………………………………………………					

* Tel: Technology enhanced learning
