# Supplementary material for: Technology Integration in Syrian Medical Education From the Perspective of Students and Faculty: A Cross-Sectional Evaluation
Source: JMIR Form Res. 2025 Aug 8;9:e76958. doi: 10.2196/76958 (PMC12334109; doi:10.2196/76958)
Supplement: Multimedia Appendix 2 [file formative-v9-e76958-s002.pdf]

A questionnaire evaluating Technology integration in Syrian medical education from the perspectives of medical college faculty

Dear participant,

Integrating technology in medical education aims to create an integrated educational system that seeks to provide educational content using modern techniques and Internet, which helps the student or learner interact with the educational material at the time, place, and speed that suits him and his circumstances and abilities, and takes into account individual differences among learners, This questionnaire was prepared for research purposes and aims to evaluate Technology integrating in medical education at Syrian universities from the perspectives of medical college faculty members and educational bodies.

We inform you that by completing this questionnaire you are totally agreeing to be part of our research, we also confirm that your participation is completely voluntary, and The collected information will be treated with complete confidentiality and will only be used for the purposes of the study. Please, write and tick where appropriate…

We are grateful for your cooperation...

Gender:
o Male o female

Specialization:
o Human medicine o Dentistry o Pharmacy o Health sciences

Academic Qualification:
o degree o Master o Ph.D

Administrative Position:
o yes o No

year of experience
. o Less than 5 years o 5-10 years o more than 10 years

	personal opinions about technology integration	Strongly Agree	Agree	Neutral	Disagree	Strongly Disagree
section.1	1.Integrating technology in medical education is a necessity nowadays					
	2.Acquiring knowledge through traditional learning is more effective*					
	3.Integrating technology in education helps acquire clinical skills better					
	4.Technology-enhanced environments restrict the role of the teacher					
	5.Technology-enhanced environments promote collaboration and teamwork					
	6.Technology-enhanced environments enhance student-centered learning					
	7.Technology-enhanced environments enhance communication skills in students					
	8.Technology-enhanced environments do not consider individual differences among students*					
	9.Technology-enhanced environments help develop self-learning and continuous learning skills					
	10.Technology-enhanced environments are more effective than traditional environments					
	11.Traditional learning approach is more applicable in our universities					
section.2	Requirements of TEL environments	Strongly Agree	Agree	Neutral	Disagree	Strongly Disagree
	12.Technology-enhanced environments do not require significant time and effort*					
	13.Integrating technology in medical education requires institutional support at all levels					
	14.Integrating technology in medical education requires support from department heads and deans					
	15.Integrating technology in medical education requires faculty training					
	16.Integrating technology in medical education does not require new teaching methods*					
	17.Traditional assessment methods are effective in technology-enhanced environment					
Section.3	challenges face Technology integration	Strongly Agree	Agree	Neutral	Disagree	Strongly Disagree
	18.the lately conditions hinder the process of integrating technology in education					
	19.Weak infrastructure and services of medical colleges hinder the integration of technology in medical education					
	20.Lack of teaching staff experience makes integration difficult					
	21.lectures time is insufficient to use modern means and technologies					
	22.Classroom facilities in colleges are adequately equipped for the integration of technology in medical education*					
	23.Technology can be applied without radical changes in the curriculum					
	24.It is difficult to apply technology with all study subjects					
	If you have any other recommendations or suggestions related, please write it down: ……………………………………………………………………………………………………					

* Tel: Technology enhanced learning
